# A New Method for Next-Generation Sequencing of the Full Hepatitis B Virus Genome from A Clinical Specimen: Impact for Virus Genotyping

**DOI:** 10.3390/microorganisms8091391

**Published:** 2020-09-11

**Authors:** Flavia Hebeler-Barbosa, Ivan Rodrigo Wolf, Guilherme Targino Valente, Francisco Campello do Amaral Mello, Elisabeth Lampe, Maria Inês de Moura Campos Pardini, Rejane Maria Tommasini Grotto

**Affiliations:** 1 Medical School, São Paulo State University (Unesp), Botucatu 18618-687, Brazil; flavia.hb.trovao@unesp.br (F.H.-B.); ines.pardini@unesp.br (M.I.d.M.C.P.); 2 Molecular Biology Laboratory of Clinical Hospital of Botucatu (HCFMB), Botucatu 18618-687, Brazil; 3 School of Agriculture, São Paulo State University (Unesp), Botucatu 18618-687, Brazil; ivanr.wolf@gmail.com (I.R.W.); valentegt@fca.unesp.br (G.T.V.); 4 Laboratory of Viral Hepatitis, Oswaldo Cruz Institute, FIOCRUZ, Rio de Janeiro 21040-900, Brazil; fcamello@gmail.com (F.C.d.A.M.); elisabeth.fiocruz@gmail.com (E.L.)

**Keywords:** Hepatitis B virus, NGS, genotyping, phylogeny analysis

## Abstract

Hepatitis B virus (HBV) is an enveloped virus that induces chronic liver disease. HBV has been classified into eight genotypes (A–H) according to its genome sequence by using Sanger sequencing or reverse hybridization. Sanger sequencing is often restricted to analyzing the S gene and is inaccurate for detecting minority genetic variants, whereas reverse hybridization detects only known mutations. Next-generation sequencing (NGS) is a robust tool for clinical virology with different protocols available. The objective of this study was to develop a new method for the study of viral genetic polymorphisms or more accurate genotyping using genome amplification followed by NGS. Plasma obtained from five chronically infected HBV individuals was used for viral DNA isolation. HBV full-genome PCR amplification was the enrichment method for NGS. Primers were used to amplify all HBV genotypes in three overlapping amplicons, following a tagmentation step and Illumina NGS. For phylogenetic analysis, sequences were extracted from the HBVdb database. We were able to amplify a full HBV genome; further, NGS was shown to be a robust method and allowed better genotyping, mainly in patients carrying mixed genotypes, classified according to other techniques. This new method may be significant for whole genome analyses, including other viruses.

## 1. Introduction

Hepatitis B is a potentially life-threatening liver infection caused by the hepatitis B virus (HBV), which can cause chronic infection. HBV is a major global health problem, and around 257 million people live with hepatitis B virus infection (defined as being hepatitis B surface antigen positive), resulting in 887,000 deaths (2015) mainly from complications, including cirrhosis and hepatocellular carcinoma [1].

HBV is an enveloped virus that belongs to the Hepadnaviridae family with a circular and partially double-stranded DNA genome of ~3.2 kb, depending on the genotype. The HBV genome structure and organization comprise four overlapping open reading frames (ORFs) with four major genes, designated as pre-S/S, C, P, and X. The pre-S1 and pre-S2 genes encode the hepatocyte receptor-binding site, whereas the S (surface) gene encodes the hepatitis B surface antigen (HBsAg). The C (core) gene encodes the hepatitis B core antigen (HBcAg) and the hepatitis B e antigen (HBeAg). The P (polymerase) gene encodes the enzymes reverse transcriptase (RT), RNase-H, and DNA polymerase. The X gene encodes a small regulatory protein which activates transcriptional promoters that are important for HBV replication [2,3]. The eight HBV genotypes (A–H) are classified by genomic divergence of more than 7.5% within each genetic group [4,5,6,7]. More recently, two additional genotypes (I and J) were proposed [8,9].

Reverse hybridization (INNO-LiPA) presents high sensitivity to detect mutations in 5% of the circulating viral population; however, only known mutations can be found [10,11]. In a Brazilian multicenter study we identified unusual mixed genotypes detected by the INNO-LiPA technique but not confirmed by direct sequencing [12]; moreover, we proposed further investigation by next-generation sequencing (NGS) to confirm the INNO-LiPA assay.

NGS is a powerful tool in clinical virology [13] and can be used to detect virus drug resistance mutations or mixed genotypes and quasispecies of HBV, as Sanger sequencing can be inefficient in detecting a patient’s minor circulating variants [11]. NGS analysis was previously used to assess the presence and outcome of genotype mixtures in the polymerase/surface and X/preCore regions of the HBV genome from patients with chronic HBV infection [14]. These authors observed genotype changes in P/S genes in four patients during natural quasispecies dynamics and in two patients during treatment. The detection of HBV drug resistance mutations in P/S genes (preferably RT polymerase) using NGS has been extensively reported [11,15,16,17]. In all those studies, pyrosequencing or Illumina was carried out after gene PCR amplification. Only one paper describes NGS analysis of full genome sequencing, although using nine amplicons [18].

The NGS protocols require minimal viral DNA concentrations for optimal results and sufficient coverage for the detection and analysis of virus variants and quasispecies. Then, a virus DNA enrichment method is necessary in clinical samples for NGS. NGS using hepatitis B clinical samples without any enrichment method generates low read numbers, low virus genome coverage, and high noise (mainly reads from the human genome). Hence, herein we propose a full hepatitis B genome amplification step as a sample enrichment method, using only three overlapping amplicons and a tagmentation step with the Nextera XT kit. The new method uses a lower amplicon number than previous studies for genome sequencing [18], presents satisfactory sequence quality, and follows an easier methodology that can be applied to sequence all HBV genotypes using the MiSeqIllumina platform.

## 2. Materials and Methods

### 2.1. Patients and Virus Specimens

Five clinical virus specimens were obtained from chronically infected HBV individuals with viral load detected by real-time polymerase reaction (Abbott Real Time HBV assay). The initial viral loads ranged from 894 to 1.4 × 10^6^ IU/mL (Table 1). The samples were genotyped by INNO-LiPA^®^ HBV Genotyping assay (Fujirebio Europe N.V., Ghent, Belgium), and four mixed infections were included in this study, specifically, mixed infections of genotypes D/G, an unusual profile of infection in Brazil. The patients were followed up in the Molecular Biology Laboratory, Clinical Hospital Botucatu Medical School, São Paulo, Brazil or in the viral Hepatitis Laboratory at Oswaldo Cruz Institute/Fiocruz, Rio de Janeiro, Brazil.

### 2.2. Ethics Approval and Consent to Participate

For the HBV samples, ethical approval was obtained from FIOCRUZ (Oswaldo Cruz Foundation, Rio DE Janeiro, Brazil) on 16 December 2013, with reference number 495.687, located in Rio de Janeiro, Brazil. Written informed consent was obtained from all patients.

### 2.3. Sample Processing

Nucleic acid was obtained by using 356μL of the original clinical specimen from clinical samples (plasma). Samples (500 μL) were centrifuged at 12,000× *g* for 5 min at room temperature, in order to avoid cellular debris in the next steps. To eliminate extracellular human DNA, 356 μL of each plasma sample was treated with 40 μL of DNAse buffer and 8U of Turbo DNAse (Turbo DNA-Free™ Kit (Ambion, ThermoFisherScientific, Waltham, MA, USA), in a final volume of 400 µL, which was centrifuged for 30 min at 37 °C. The digestions were stopped by adding 40 µL of DNase inactivation reagent, mixing, and incubating for 5 min at room temperature. After centrifugation at 10,000× *g* for 1.5 min, the plasma was transferred to a fresh tube and immediately used for DNA extraction.

### 2.4. DNA Extraction and qPCR

Viral DNA was extracted using the QIAamp DNA Blood Mini Kit (QIAGEN, 51104), and the following changes were made: 400 μL of processed sample (previously described) was extracted, and 40μL of proteinase K, 400 μL of buffer AL, a 400 μL volume of ethanol for washing, and, finally, 50 μL of water for elution were used. The DNA concentration was analyzed using a fluorometer (Qubit 2.0, Invitrogen, Carlsbad, CA, USA) with a DNA High Sensitivity Assay kit (Invitrogen, Carlsbad, CA, USA). For all clinical samples, DNA was used as the template for qPCR reactions to verify whether DNAse treatment can affect viral DNA amplification, according to an in-house real- time PCR assay with some modifications [19]. The qPCR reaction using GoTaq^®^qPCR Master Mix (Promega, A6001) was performed on an Applied Biosystems 7300 Real-Time PCR System (Applied Biosystems, Foster city, CA, USA) in a 20 μL reaction solution containing 10μL master mix, 0.7 μL of each forward primer (F1primer 5′-CAACCTCTTGTCCTCCAACTTGT-3′; F2 primer 5′-AACCTCCTGTCCTCCAACTTGT-3′ and F3 primer 5′-CAACCTGTTGTCCTCCAATTTGT-3′), 2 μL of reverse primer (R primer 5′-GATGAGGCATAGCAGCAGGAT-3′), and 5 μL of DNA. The cycling conditions were 50 °C for 2 min, 95 °C for 2 min, 45 cycles of 95 °C for 15 s, followed by 60 °C for 1 min. As a reference standard for the viral loads, four HBV samples from the diagnostic routine were used to construct the standard curve.

### 2.5. Primer Design and Full-Genome PCR Reactions

For all five clinical samples with low or high viral loads, the DNA was used as the template for full-genome PCR reactions before Illumina NGS. Full-length genome sequences representing each one of the eight HBV genotypes were downloaded from the Hepatitis B Virus Database [20,21] and used for primer design. Primers (Table 2) were manually designed in conserved regions to amplify all HBV genotypes in three overlapping amplicons denominatingthe A (1460 bp), B (829 bp), and C (1208 bp) regions.

The primer specificity was evaluated using a Blast algorithm over the NCBI database. To ensure primer sequence identity with the viral variants, degenerate bases were inserted when necessary throughout primers, the exceptions being the last three nucleotides at the 3′ end (Cs or Gs were present at the 3′ end). PCR was carried out using the high-fidelity platinum Taq DNA polymerase (Life Technologies, Carlsbad, CA, USA) according to the manufacturer’s instructions, with some modifications for each genome region, with1.5, 2.0, and 1.5 mM of added MgSO4 for the A, B, and C regions, respectively, and 0.2, 0.2, and 0.5 μM of each primer for the regions A, B, and C, respectively. Moreover, 10 μL of the DNA, 1× High Fidelity PCR Buffer, 200 μM deoxynucleoside triphosphates (dNTP), and 1U Taq DNA polymerase (Invitrogen) were added for all reactions. The cycling conditions were 1 min at 94 °C for all regions, followed by 25 cycles of 30 s at 94 °C; 1 min at 52 °C for region A, 30 s at 55 °C for region B, and 1 min at 61 °C for region C; and 2 min at 68 °C. A final elongation of 7 min at 68 °C was done for all samples. The nested PCR conditions were the same as the previous PCR reactions but the amplicons used as the template and the cycling conditions were adjusted, when necessary, according to initial viral load: 1—for amplicon A, the PCR product was adjusted to 5 μL for samples with high viral load and 15 PCR cycles; 2—for amplicon C, the PCR product was adjusted to 20 μL for samples with low viral load and 40 PCR cycles. It was not necessary to adjust for amplicon B. The nested PCR products (8 μL) were checked by 1% agarose gel electrophoresis with GelRed™ staining (Biotium, 41003) and further purified using Invisorb^®^ Fragment Clean Up (Stratec Molecular GmbH, Berlin), according to the manufacturer’s instructions. The purified nested PCR reactions were quantified by commercial fluorometer (Qubit 2.0, Invitrogen, Carlsbad, CA, USA) with a DNA High Sensitivity Assay kit (Life Technologies1, Q33120).

### 2.6. Next-Generation Sequencing

For each sample the three PCR templates (A, B, and C) were adjusted to 0.2 ng/μL and pooled in equimolar concentration. Library preparation was performed using the Nextera XT DNA Sample Preparation Kit (Illumina, FC-131-1024), accordingly to the manufacturer’s instructions, with the modification of 0.5 ng DNA input (pool of the A, B, and C regions for each sample). According to the Nextera XT kit, using the maximum amount of recommended input (1 ng DNA) ensures libraries with high-quality sequencing results. However, when we used the maximum concentration, we observed that the average library size was larger than expected after tagmentation; thus, half of the DNA input was used in this protocol. After PCR indexing, libraries were purified with 90 μL of magnetic beads, according to the manufacturer’s instructions (AgencountAMPure XP; Beckman Coulter, A63881). Libraries were diluted to 1:10,000 and quantified using the KAPA SYBR Fast Universal qPCR Kit (KapaBiosystems, KK 4824). The kapa product (5 μL) was submitted to electrophoresis in a 2% agarose gel stained with GelRed™ (Biotium, 41003) to assess average library fragment sizes. Library quantification was performed according to Equation (1):

(1) $$C=\frac{\frac{452.X.D}{1000}}{L}$$

where *C* is the concentration in nM, 452 is a constant (KAPA fragment size in bp), *X* is the concentration in pM obtained with KAPA (qPCR result for each library), *D* is a dilution, and *L* is the library size (the approximated fragment sizes of each library according to agarose electrophoresis).

In the next step, the libraries were manually normalized and the concentration of each library was adjusted to 4 nM prior to library pooling. To ensure the library concentration, all samples were re-quantified with KAPA and re-diluted if necessary. The library pool was denatured and diluted at a final concentration of 12 pM according to the manufacturer’s instructions (Preparing DNA Libraries for Sequencing, Miseq Guide). The PhiX control library (PhiX Control Kit v3, FC-110-3001) at 10% was used as a sequencing control. Libraries were sequenced with MiSeq Reagent kit V2 300 cycles (paired 2 × 150 bp).

### 2.7. NGS Data Processing, Mapping, and Genome Comparisons

The analyzed data was deposited in the NCBI SRA database under the bioproject number PRJNA659786 and the accession numbers SRR12535947, SRR12535946, SRR12535938, SRR12535937 and SRR12535936. The read quality was accessed using FastQC and reads of <150 nt were excluded using Trimmomaticv0.36 [22]; the latter program was also used to filter the reads (Illuminaclip, Headcrop = 14, Leading = 3, Trailing = 3, Slidingwindow = 4:30, Minlen = 18).

The high-quality reads (all samples with minimum Q-Score = 30) were mapped, independently, over all genomes of all eight HBV genotypes obtained from HBVdb [21] using Bowtie2 v2.3.2. [23] with stringent parameters (–very-sensitive, –no-discordant –N 0 –L 4, –gbar 10). The aligned read counting was performed using Bed tools multiBamCovv2.25.0 [24] (default) and was loaded into an in-house Python script for mean coverage plot and genotype determination; moreover, genomic coverage throughout the A, D, and G genomes was compared using Circos plot [25].

### 2.8. Phylogenetic Analysis

Whole genomes and sequences related to the B region (genomics coordinates ~465–1328) for all genotypes were extracted from HBVdb. We selected as the outgroup the Woolly monkey hepatitis B virus clone WMHBV-2 (NCBI Access number NC_028129.1) genome and B region. Representative sequences for each set (genome or B region) were independently obtained by applying CDHIT v4.6 [26] (-s 0.95, -aL 0.95, -AL 100, -aS 0.95, -AS 100, -uL 0.50, -uS 0.50, -U 100) and aligned with MAFFT [27] (default) (Figures S1 and S2). The jModeltest [28] wasused to fit the best evolutionary model (searched using Bayesian information criterion) for both alignments. The phylogenetic inferences by maximum likelihood were obtained by RaxML [29] (-T 10, -f a, -m GTRGAMMAI, -p 12345, -x 12345, -# 100) and bootstrap information with -T 10, -f, b, -m, GTRGAMMAI.

## 3. Results

### 3.1. DNA Quantification and qPCR

Quantification of DNA after DNAse treatment and extraction showed negative results in the fluorometer Qubit 2.0, even considering samples with high viral load and using a high-sensitivity assay kit. However, all samples were positive in an in-house real-time PCR assay with viral loads above 2.000 UI/mL, showing that DNAse treatment did not affect viral DNA amplification. Moreover, DNAse treatment did not change viral load quantification by qPCR (Table 1).

### 3.2. Full-Genome Amplification by NestedPCR

**Figure 1:**
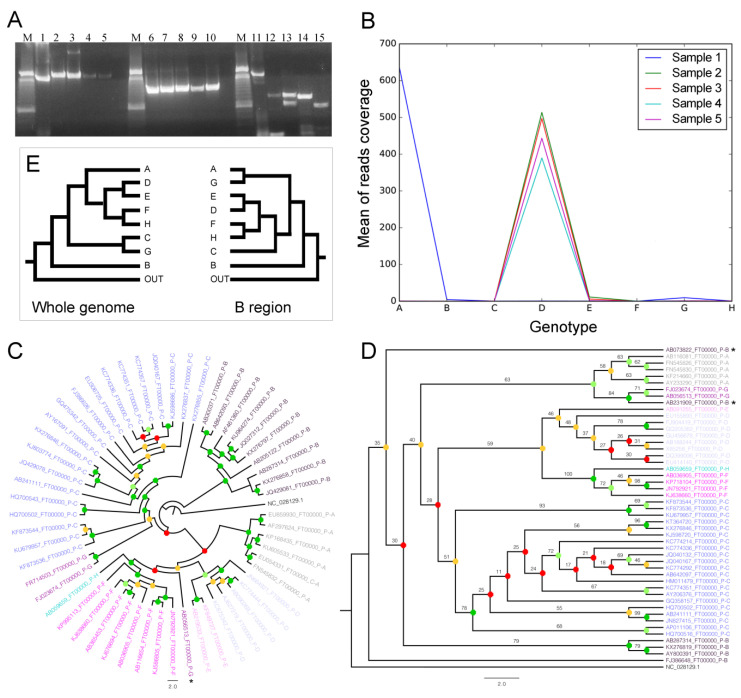
Electrophoresis, mapping, and phylogenetic trees (cladograms): (**A**) Hepatitis B virus (HBV) genome amplification from clinical samples. Amplicons from the A, B, and C regions were checked using an agarose gel stained with GelRed™. M represents the DNA Ladder 100 bp (Invitrogen, cat.15628-050). The strong reference band represents 600 bp. Lanes 1 to 5 represent amplicons from the A region. Lanes 6 to 10 represent amplicons from the B region and lanes 11 to 15 represent amplicons from the C region; (**B**) Mean of reads mapped over major HBV genotypes; (**C**,**D**) Phylogenetic trees inferred using whole genomes and using the B region, respectively. Bootstrap was binned with ranges 0–30, 31–60, 61–75, and 75–100, indicated by the red, orange, light-green, and dark- green circles, respectively. Bar, 0.2 modifications; (**E**) Synthesis of phylogenetic trees showing only genotype grouping.

The nested PCR assay was able to generate amplicons from all clinical samples, even those with the lowest viral load. The three overlapping amplicons (A–B–C) checked by electrophoresis are shown in Figure 1. Using a Blast algorithm, all primers showed specificity for HBV, without any similarity to genomes from other organisms (like *Homo sapiens* or *Escherichia coli*). This assay showed high sensitivity and specificity, and it can be used for genome amplification of any clinical sample with viral load detected in routine laboratory assays. The dsDNA concentration of each nested PCR amplicon was between 3 and 900 ng/μL, analyzed by a fluorometer. The C-amplicon showed the lowest concentrations of DNA in the fluorescence assay, as this region was more difficult to amplify, so the viral load should be >100 IU/mL and freezing and thawing of the samples should be avoided. The C region amplified from samples 2–5 presented a length of less than 1208 bp due to a genome deletion, but this did not interfere with genotyping results.

### 3.3. Library Quantification

All libraries were quantified successfully with CT values ranging from 9 to 12, within KAPA standard curve values. Libraries showed a broad size distribution of 250–700 bp (Figure S3), and library average fragment sizes were defined as 350 bp. According to the quantification using Equation (1), libraries showed molar concentrations between 12 and 94 nM. Illumina recommends manual normalization when the library yield is less than 10–15 nM, which was performed here.

### 3.4. NGS Data Analyses and Genome Coverage

#### 3.4.1. Cleaning

On average, around ~35.41% of reads were maintained after quality filtering (Table 3) as we used a stringent filtering protocol, giving us only the high-quality regions of the reads (Q-Score of ≥38 for all positions) (Figure S4).

#### 3.4.2. Genomic Mapping, Comparisons, and Genotyping

Read mapping produced a range from 80.24% to 96.76% for the overall alignment rate. The average of raw reads mapped on all genomes from all eight HBV genotypes shows that most of the reads for sample 1 accumulated over genotype A, while a minor amount of reads were in B and G. For samples 2, 3, 4, and 5, most of the reads were mapped over genotype D, while a minor amount of reads were over genotype E for samples 2 and 3 (Figure 1B). Thus, the read mapping on the specific genotypes can indicate which genotypes are present in each sample since we used rigorous alignment parameters (see the topic “NGS data processing, mapping, and genome comparisons” in “Materials and Methods”).

**Figure 2:**
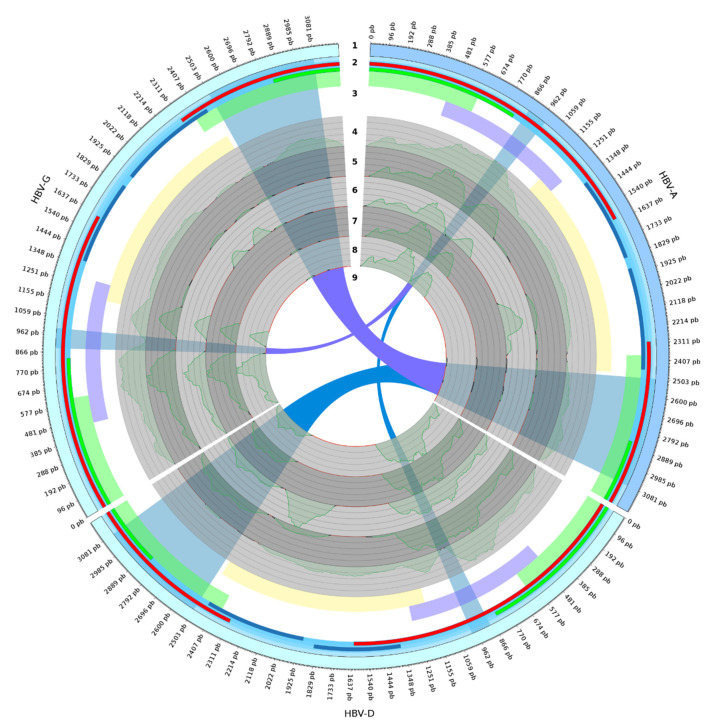
Diagram showing three HBV genotypes, amplicons, and mapping results. The 12 o’clock numbers in rings indicate (**1**) genome extension; (**2**) annotations, where red is the two regions of gene P (Polymerase; 2307–3215 nt and 1–1623 nt) and greens are the two regions of gene S (Gene PreS1, PreS2 and Gene HBsAg; 2848–3215 nt and 1–835 nt); (**3**) the three amplicon regions, where lightgreen, lightpurple, and lightyellow are the A, B, and C amplicons, respectively; (**4**–**8**) raw coverage ofread alignments from samples 1–5, respectively, where red, black, and green lines mean <500, ≥500–2000, and ≥2000 reads mapped, respectively; (**9**) links between homologous regions with differential coverage among genotypes.

Once we noticed that there were a few reads mapping in the G genotype (Figure 1B), we further explored the alignments on amplicon regions (Figure 2) and constructed the phylogenetic trees. The coverage plots and phylogenetic trees allowed us to find that a putative mis-hybridization of the INNO-LiPA probe was the reason behind the mixed genotype detection in samples without the G genotype. The phylogenetic inference using the whole genomes showed that only one G genotype sequence was grouped out of its main clade, but with a very low branch support (bootstrap = 22) (Figure 1C), which means it must be a mis-grouping. Moreover, all genotypes were confidently grouped since all main clades had a high branch support (bootstrap >75) (Figure 1C). Hence, since the mappings were very sensitive, the reads mapped over B, E and G genotypes (Figure 1B) were only spurious mis-alignments, and there were only the genotypes A and D in our samples.

Interestingly, the phylogenetic tree using B regions (the region utilized by the INNO-LiPA kit) showed most of the branches with low support (bootstrap <61). Moreover, two B sequences were grouped out of their main clade, but only one was highly supported (bootstrap = 84) (Figure 1D). We can also see that both phylogenetic trees have completely divergent groupings, with the only convergence being the B genotype as a sister group of all other genotypes (Figure 1E), despite low support in the B region tree (Figure 1D). We can see in Figure 1D (the phylogenetic tree using only the B-region) the genotypes A and G are grouped in the same clade (resumed in Figure 1E), suggesting that these genotypes are phylogenetically closer. Genotyping using only this region may prevent correct detection of HBV minority viral variants.

The A–B–C regions (amplicons A, B, and C) in the HBV genome are shown in Figure 2, in lightgreen, lightpurple, and lightyellow, respectively.

The raw read mapping of all samples over three HBV genotypes (HBV-A, HBV-D, and HBV-G) showed two high-coverage regions only for sample 1 when mapped over the A genotype (Figure 2, in coordinate ranges ~870–965 and ~2503–3081), while showing very different coverage when mapped over the D genotype (sample 1 has the lowest coverage). Interestingly, the same coverage pattern was present for the second region when the reads were mapped over genotype A and G, while region 1 had low coverage for all samples over the G genotype.

We suppose that co-circulating A and G can constitute a technical limitation based on observation of samples 2, 3, and 5. The genotyping using reverse hybridization detected mixtures D/G (Table 1), and the NGS method showed only the D genotype, which corroborates the mappings (Figure 2). The lack of specificity was due to the hybridization methodology. The A region (Figure 2, light green on ring 3) of sample 1 showed mapped reads in both the A and G genotypes, but from analysis of Figure 1B, the G genotype presented coverage close to zero. We concluded that the G genotype mapping in sample 1 was due to sequence similarity in this genomic region, regardless of the phylogenomics (Figure 1C) that did not group A with G. The incorrect A/G mapping is in agreement with B-region phylogenetics (Figure 1D); however, the mis-mapping (and the consequent genotyping errors) can be avoided by mapping reads in the whole HBV genome (Figure 1B and Figure 2). The B region (region ~458–1286 nt; Table 2 Figure 2) includes 355 nt of the P/S region (615–969 nt) analyzed by Caballero et al. [14] and also the region used by INNO-LIPA for genotyping (~456–798 nt).

## 4. Discussion

A previous study performed with our research group in Brazil found a high proportion of mixed G-genotype infections, especially D/G (an unusual co-infection), according to a reverse hybridization assay [12]. However, by NGS analysis, we showed that this mixed genotype D/G was not confirmed. We performed molecular biology and bioinformatics analyses that showed that the method here proposed is useful to discriminate mixtures that have been classified as dual population in infected patients. This result was consistent with those of PCR assays to detect mixed viral populations, where the samples classified as mixed D/G or F/G infection were considered mono-infections with genotype D or F, respectively [12]. Thus, this paper confirmed our previous study and showed that D/G mixed infection does not occur in Brazil.

This is very important since in the literature, mixed infections (especially A/G and A/D) have been detected based on P/S partial gene Sanger sequencing and INNO-LiPA HBV genotyping assays [14,30,31]. S-PCR DNA product sequencing is partially effective for the detection of artificial mixed samples, and INNO-LIPA can overestimate mixed infections as a result of erroneous genotype H detection [32]. In this work, the results are in concordance with those by other authors [33] where phylogenetic trees inferred using whole genomes or alternatively using the B region (including nucleotide sequences used by INNO-LIPA) showed different results. In fact, phylogenetic trees constructed for HBV using whole genomes were reproduced only in the P-complete gene phylogenetics [33]. Methods for genotyping based only on the S gene can cause mis-mapping, and the close association between the HBV/G and HBV/A genomes due to high homology within the S gene sequence [33] may cause incorrect detection of A/G mixed infection, a usual co-infection profile. However, it is important to highlight that there was 100% agreement between NGS and INNO-LIPA regarding the detection of the predominant genotype, requiring care only in interpreting the identification of mixtures of genotypes when using hybridization methods or P/S partial gene sequencing.

Agreements between reverse hybridization and NGS results were probably due to the detection of the majority genotypes, and clashing results between techniques can be resolved by whole-genome sequencing [17].

## 5. Conclusions

Our results showed that the NGS method proposed herein is an appropriate technique for correct HBV genotyping, mainly in patients carrying mixed genotypes classified according to other techniques, and could be used in further studies to screen for resistance mutations.

In this study, we described a robust assay based on amplification of the full-length HBV genome in only three overlapping regions. This method of clinical sample enrichment for NGS was essential to obtaining satisfactory Illumina NGS results, which are useful for the correct detection of mixed viral populations or viral genetic polymorphisms.

This new NGS protocol could be useful for HBV genotyping and evaluating the circulating variants in infected patients. NGS sequencing of other genotypes using this new method is important for HBV genotyping studies. Moreover, the results from this new NGS protocol maybe useful for the identification of clinically relevant variants or for the discovery of new resistance-associated variants. Finally, this strategy for whole-genome sequencing with prior amplification and tagmentation steps can also be applied to other viruses from clinical samples.

## Figures and Tables

**Table 1:** Sample information of hepatitis B (HBV) mono-infected patients.

Sample ID	Initial Viral Load (UI/mL and Log) ^a^	Viral Load after DNAse (UI/mL and Log) ^b^	dsDNA (ng/μL) ^c^	Genotype (INNO-LiPA) ^d^
1	536,525/5.73	910,000/5.95	ND	A
2	1,439,366/6.16	1,700,000/6.23	ND	D/G
3	120,781/5.08	330,000/5.52	ND	D/G
4	894/2.95	2200/3.34	ND	A/D
5	2654/3.42	3900/3.59	ND	D/G

ND = not detected. ^a^ HBV DNA quantification using Real Time HBV Amplification Reagent Kit, Abbott (sensitivity of 10 IU/mL for 0.5 mL sample volume and specificity 100%), prior the DNAse treatment and library preparation with Nextera-XT. ^b^ in house qPCR assay after DNAse treatment. ^c^ dsDNA concentration measure using Qubit^®^ (High Sensitivity DNA Kit, ThermoFisher, Waltham, MA, USA) after DNAse treatment. ^d^ HBV genotyping kit (Fujirebio Europe N.V., Ghent, Belgium) [12].

**Table 2:** Primers for full-length genome HBV amplification.

Primers	Sequence (5′–3′)	Genome Binding Position ^a^	Amplicon Size (bp)
AF	AAG AAC TCC CTC GCC TC	2374–2390	
AR	GAT GAT GGG ATG GGA ATA CAR GTG	595–618	1460
BF	GGT ATG TTG CCC GTT TGT CC	458–477	
BR	GCW AGG AGT TCC GCA GTA TGG	1266–1286	829
CF	GCT GAY GCA ACC CCC ACT G	1186–1204	
CR	CTG CGA GGC GAG GGA GTT C	2376–2394	1208

^a^ Genome binding position (nucleotides) according to HBV genotype B complete genome(isolate: P2-121214, GenBank: AB981583.1).

**Table 3:** Basic statistics before and after cleaning for a subgroup of samples.

	Before Cleaning		After Cleaning		
Sample ID	N. R. ^a^	Sequence Range	N. R. ^a^	Sequence Range	P.S.R. ^b^
1	729,656	35–151	265,092	16–135	36.33
2	654,788	35–151	236,472	16–135	36.11
3	716,362	35–151	229,617	16–135	32.05
4	585,970	35–151	215,458	16–135	36.76
5	672,119	35–151	240,780	16–135	35.82

^a^ Number of reads; ^b^ Percentage of surviving reads.

## Supplementary Materials

The following are available online at https://www.mdpi.com/2076-2607/8/9/1391/s1, Figure S1: Genome alignment of representative sequences of HBV used to make the phylogenetic inference, Figure S2: Alignment of representative sequences of HBV B region used to make the phylogenetic inference, Figure S3: Broad size fragments from libraries after the Nextera XT procedure, Figure S4: Example of quality score before (A) and after (B) cleaning in library from sample 4.
