# Supplementary figures and images for: A New Method for Next-Generation Sequencing of the Full Hepatitis B Virus Genome from A Clinical Specimen: Impact for Virus Genotyping
Source: Microorganisms. 2020 Sep 11;8(9):1391. doi: 10.3390/microorganisms8091391 (PMC7564258; doi:10.3390/microorganisms8091391)

**600bp**

**M**


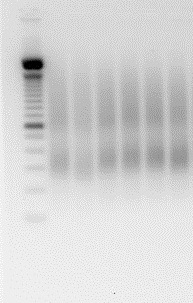


Figure S3: Broad size fragments from libraries after the Nextera XT procedure

Supplement: Supplementary file 1 [file microorganisms-08-01391-s001.zip › S3_Fig.docx]

**A**


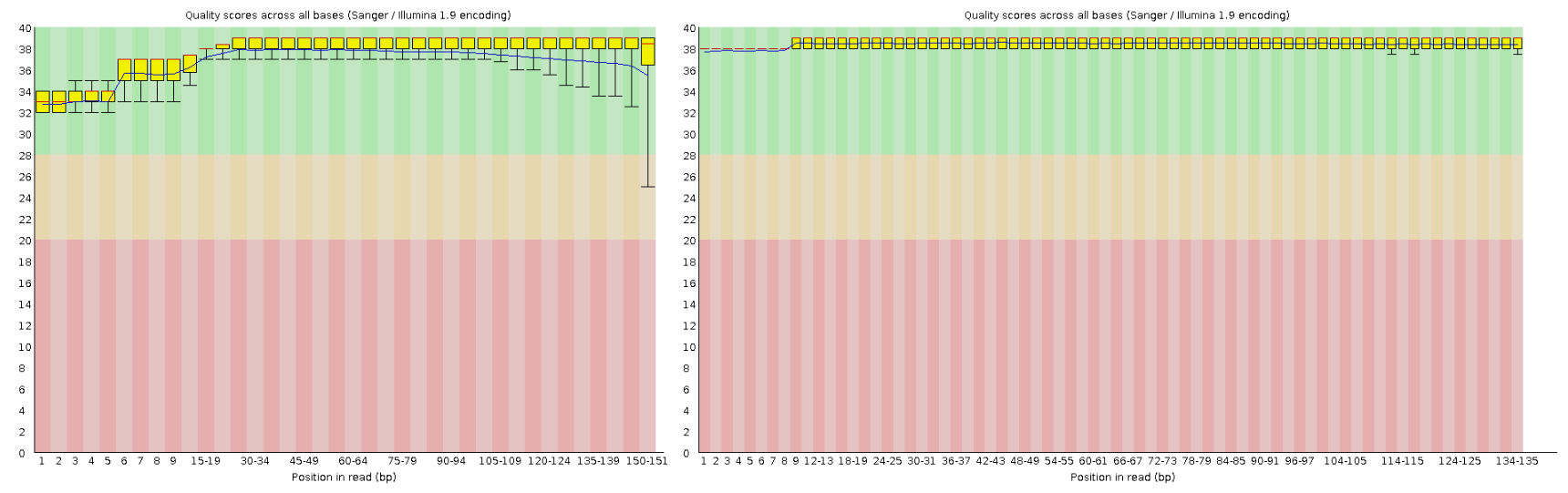


**B**


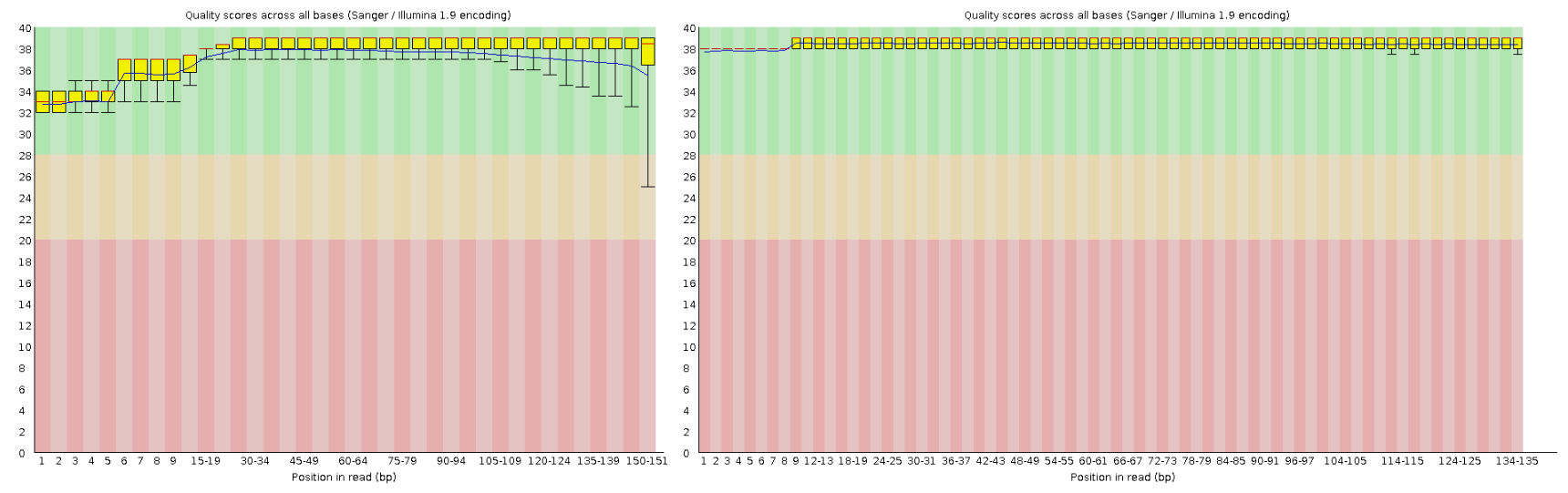


Figure S4: Example of quality score before (**A**) and after (**B**) cleaning in library from sample 4.

Supplement: Supplementary file 1 [file microorganisms-08-01391-s001.zip › S4_Fig.docx]
